# Sur la collection de phlébotomes (Diptera: Psychodidae) du Professeur Jean-Antoine Rioux

**DOI:** 10.48327/mtsibulletin.2021/127

**Published:** 2021-09-23

**Authors:** J. Depaquit

**Affiliations:** 1 Université de Reims Champagne Ardenne, ESCAPE EA7510, USC ANSES VECPAR, SFR Cap Santé, UFR de Pharmacie, 51097 Reims, France.

**Keywords:** Collection, Phlébotomes, centre de ressources biologiques, *Phlebotomus*, *Sergentomyia*, *Grassomyia*, France, Italie, Espagne, Algérie, Tunisie, Maroc, Yémen, Syrie, Soudan, République du Congo, Collection, Phlebotomine Sand flies, Curated collection, *Phlebotomus*, *Sergentomyia*, *Grassomyia*, France, Italy, Spain, Algeria, Tunisia, Morocco, Yemen, Syria, Sudan, Republic of the Congo

## Abstract

**Introduction:**

Le Professeur Jean-Antoine Rioux (1925-2017) a passé une grande partie de sa longue et fructueuse carrière scientifique à explorer et comprendre le fonctionnement de foyers de leishmanioses dans plusieurs régions de Méditerranée, du Proche et du Moyen-Orient et d'Afrique. L’étude des phlébotomes constitua une partie importante de son travail dans les foyers étudiés et un grand nombre de spécimens ont été mis en collection, objet de cette note.

**Matériel et Méthode:**

La collection a été complètement inventoriée. Les phlébotomes y sont classés par espèces et par pays. Chaque phlébotome est monté *in toto* individuellement sur une lame dans du baume du Canada.

**Résultats:**

La collection est riche de 130 840 spécimens provenant de 10 pays: France (continentale et Corse), Italie, Espagne, Algérie, Tunisie, Maroc, Yémen, Syrie, Soudan et République du Congo. Elle regroupe 26 espèces: *Phlebotomus alexandri, Ph. ariasi, Ph. bergeroti, Ph. chabaudi, Ph. chadlii, Ph. kazeruni, Ph. longicuspis, Ph. mascittii, Ph. mongolensis, Ph. orientalis, Ph. papatasi, Ph. perfiliewi, Ph. perniciosus, Ph. riouxi, Ph. sergenti, Sergentomyia africana, Se. antennata, Se. christophersi, Se. clydei, Se. fallax, Se. minuta, Se. schwetzi, Se. silva, Se. taizi, Se. tiberiadis* et *Grassomyia dreyfussi.*

**Discussion:**

La collection des phlébotomes de Jean-Antoine Rioux (1925-2017) a été transférée dans la Plateforme des centres de ressources biologiques de Reims (PF CRBs Reims) où elle est entretenue et inventoriée afin d’être mise à la disposition des personnes désireuse d'y accéder à toutes fins scientifiques. Outre des topotypes de *Ph. chabaudi* et de *Ph. chadlii,* elle regroupe de nombreux exemplaires d'Afrique du Nord, essentiellement du Maroc, mais également des exemplaires de Syrie ou du Yémen dont l'intérêt est évident compte tenu du contexte géopolitique actuel.

## Abstract (en)

**Background:**

Professor Jean-Antoine Rioux (1925-2017) has spent much of his long and fruitful scientific career exploring and understanding the functioning of leishmaniasis foci in several regions of the Mediterranean, the Near and Middle East and Africa. Phlebotomine sand flies study was an important part of his work in the foci studied and a large number of specimens have been collected by himself and stored in his collection, detailed in this note.

**Materials and Methods:**

His collection has been completely inventoried. Phlebotomine sand flies have been classified here by species and by country. Each specimen is mounted individually *in toto* between slide and cover-slip in Canada balsam.

**Results:**

The collection includes 130 840 specimens coming from 10 countries: France (mainland and Corsica), Italy, Spain, Algeria, Tunisia, Morocco, Yemen, Syria, Sudan, and Republic of Congo. These specimens belong to 26 species: *Phlebotomus* (*Artemievus*) *alexandri, Ph.* (*Larroussius*) *ariasi, Ph.* (*Phlebotomus*) *bergeroti, Ph.* (*Paraphlebotomus*) *chabaudi, Ph.* (*Lar.*) *chadlii, Ph.* (*Par.*) *kazeruni, Ph.* (*Lar.*) *longicuspis, Ph.* (*Transphlebotomus*) *mascittii, Ph.* (*Par.*) *mongolensis, Ph.* (*Lar.*) *orientalis, Ph.* (*Phl.*) *papatasi, Ph.* (*Lar.*) *perfiliewi, Ph.* (*Lar.*) *perniciosus, Ph.* (*Par.*) *riouxi, Ph.* (*Par.*) *sergenti, Sergentomyia* (*Parrotomyia*) *africana, Se.* (*Sergentomyia*) *antennata, Se.* (*Sintonius*) *christophersi, Se.* (*Sin.*) *clydei, Se.* (*Ser.*) *fallax, Se.* (*Ser.*) *minuta, Se.* (*Ser.*) *schwetzi, Se.* (*Ser.*) *silva, Se.* (*Ser.*) *taizi, Se.* (*Sin.*) *tiberiadis* and *Grassomyia dreyfussi.*

**Discussion:**

The collection was transferred to the Platform of Reims Biological Resource Centers (PF CRBs Reims) located at the Biology Department of the Reims University Hospital in order to be curated and stored physically and electronically in a secure manner. In a near future, it will be made available to scientists wishing to access it for all purposes.

In addition to *Ph. chabaudi* and *Ph. chadlii* topotypes, the collection includes many specimens from North Africa, mainly from Morocco (53% of the collection is originated from this country where Professor Rioux worked during 30 years) but also specimens from Syria or Yemen, the interest of which is obvious given the current geopolitical context.

## Introduction

Durant toute sa carrière, le Professeur Jean-Antoine Rioux a consacré une grande partie de ses travaux à l’étude des leishmanioses en procédant toujours à l’échantillonnage concomitant de tous les acteurs des cycles: phlébotomes vecteurs, hôtes vertébrés (réservoirs animaux et patients) et parasites (*Leishmania* spp) [16, 17, 18].

Ayant toujours cherché à travailler les foyers leishmaniens de manière exhaustive, le volet entomologique des études qu'il a dirigées ou auxquelles il participait a toujours été très bien développé. Les stratégies d’échantillonnage mises en œuvre couplaient toujours des méthodes complémentaires (pièges adhésifs, pièges lumineux de type CDC, captures manuelles nocturnes). Ce volet comprenait :

- le montage individuel d'un seul spécimen par lame;
- l'identification de tous les spécimens capturés;
- la mise en collection de tous les spécimens.

Quelques semaines avant son décès, J.-A. Rioux a souhaité rencontrer l'auteur de cette note afin de lui réitérer son désir de lui léguer sa collection de phlébotomes.

Cet article détaille avec précision le contenu de cette collection, son lieu de dépôt et la manière pour les collègues intéressés d'y accéder.

## Matériel et Méthode

**Figure 1:**
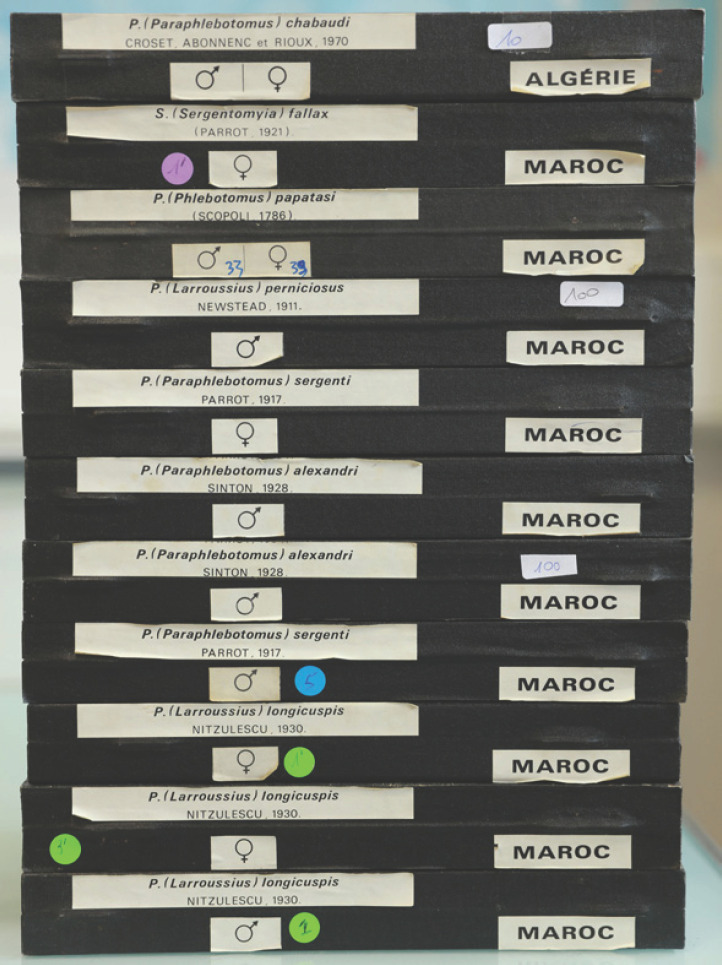
Boites de lames de la collection de J.-A. Rioux ©J. Depaquit Boxes of microscopic slides of J.-A. Rioux’ collection ©J. Depaquit

**Figure 2:**
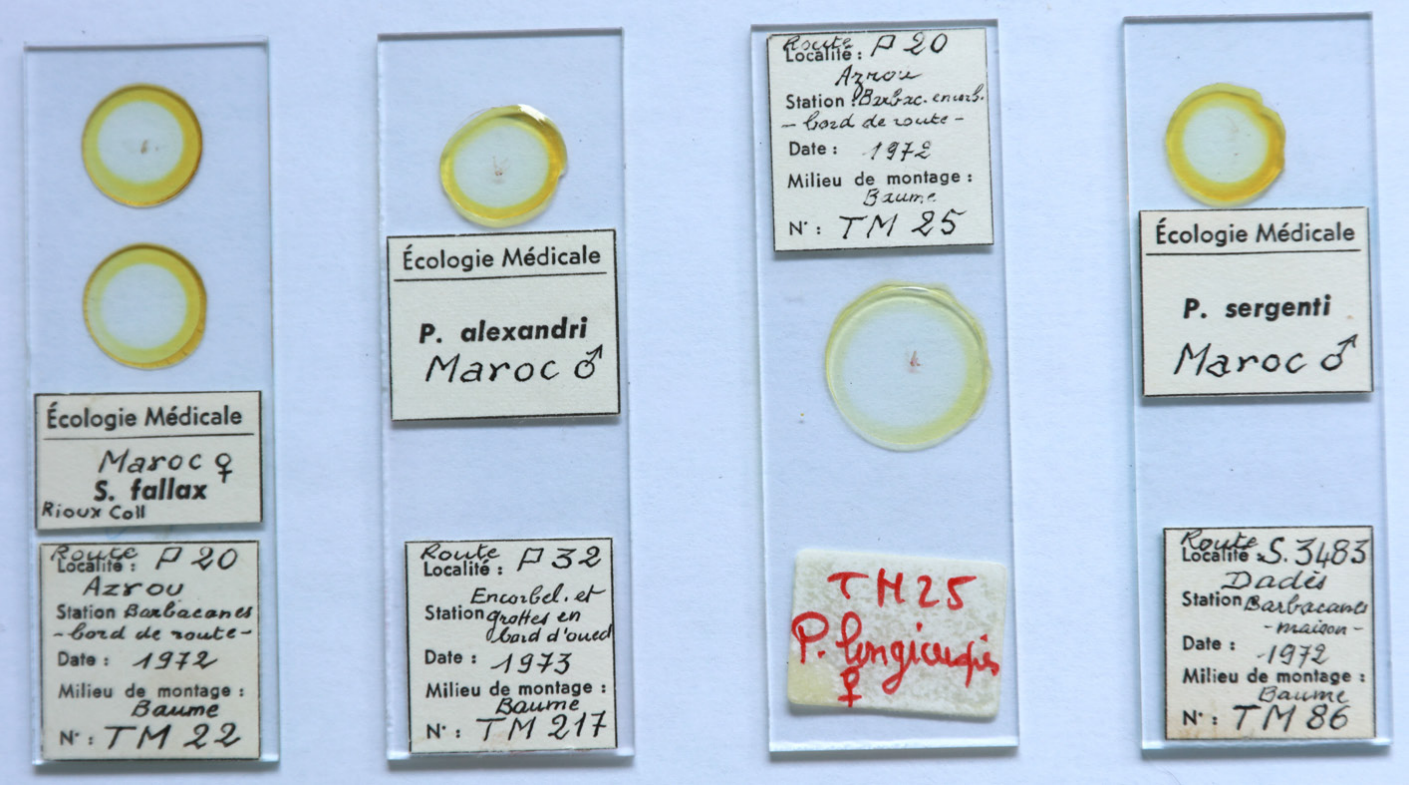
Lames de la collection de J.-A. Rioux ©J. Depaquit Microscopic slides of J.-A. Rioux collection ©J. Depaquit

Un inventaire minutieux de la collection a été réalisé. Les phlébotomes y sont rangés par espèces et par pays dans leurs boites d'origine (Fig. 1), sauf exception de boites abîmées qui ont alors été remplacées. Chaque phlébotome est monté *in toto* individuellement dans du baume du Canada entre lame et lamelle. Les lames sont gravées et beaucoup possèdent leur étiquetage d'origine (Fig. 2).

L'inventaire a été réalisé par un examen minutieux de chaque boite et par un inventaire soigneux des lames qu'elle contenait.

## Résultats

**Figure 3:**
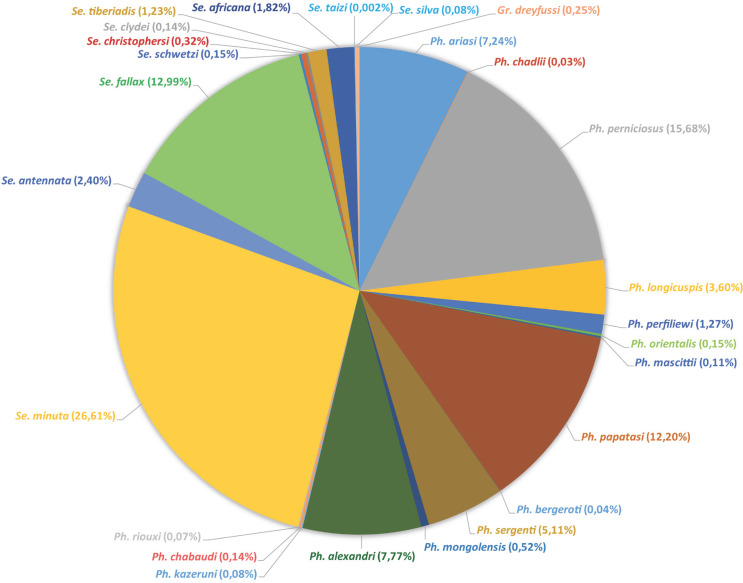
Ventilation des phlébotomes de la collection de J.-A. Rioux par espèces Relative abundance of species included in J.-A. Rioux’ collection

La collection est riche de 130 840 spécimens se divisant entre 68,7 % de mâles et 31,3 % de femelles. Ils appartiennent à 26 espèces différentes à savoir 15 espèces du genre *Phlebotomus,* 10 du genre *Sergentomyia* et 1 du genre *Grassomyia* (Fig. 3).

**Figure 4:**
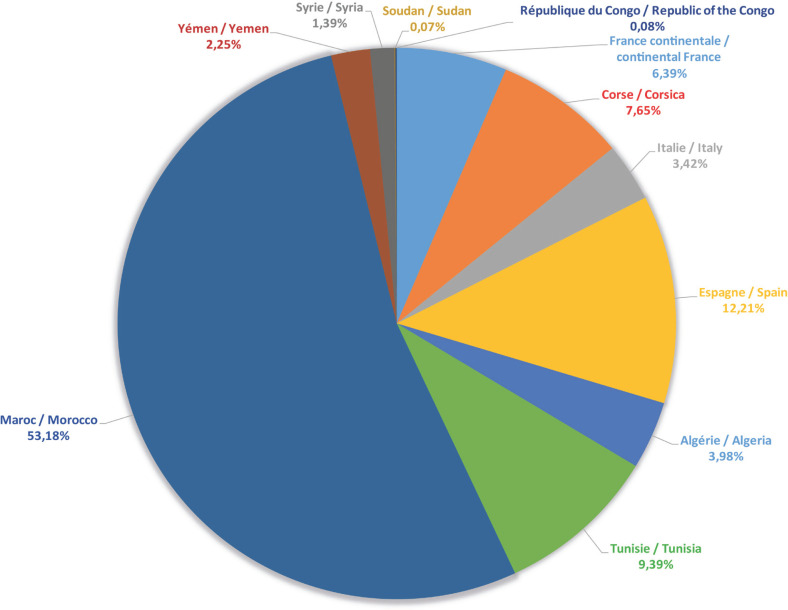
Ventilation des phlébotomes de la collection de J.-A. Rioux par pays ou par région de capture Countries and areas where the sand flies included in J.-A. Rioux’ collection have been sampled

Ces phlébotomes proviennent de 10 pays différents. En ce qui concerne les phlébotomes de France, nous avons séparé les exemplaires de France continentale de ceux de Corse pour des raisons biogéographiques évidentes (Fig. 4).

## Discussion

J.-A. Rioux était un homme de terrain. Les très nombreuses missions auxquelles il a participé, qu'il a souvent dirigées, et son grand intérêt pour la mise en collection expliquent la richesse de sa collection de phlébotomes (Tableau I). Le nombre beaucoup plus grand de mâles présents dans sa collection que de femelles s'explique par l'objectif épidémiologique de bon nombre de missions visant à la caractérisation des vecteurs dans de nombreux foyers qui nécessitait la dissection des femelles capturées sur le terrain en vue de l'isolement de leishmanies.

**Tableau I:** inventaire de la collection de phlébotomes de J.-A. Rioux Specimens included in the collection of J.-A. Rioux

		France continentale	Corse	Italie	Espagne	Algérie	Tunisie	Maroc	Yémen	Syrie	Soudan	République du Congo	Total par sexe	Total par espèce
*Phlebotomus ariasi*	♂ ♀	3517 649			3359 723		10	1093 126					7979 1498	9477
*Ph. chadlii*	♂ ♀						29	15					44 0	44
*Ph. perniciosus*	♂ ♀	775 148	6615 746	1340	6196 1464	2416		774 41					18116 2399	20515
*Ph. longicuspis*	♂ ♀					292	292	3825 305					4409	4714
*Ph. perfiliewi*	♂ ♀			125 4		1168	369						1662 4	1666
*Ph. orientalis*	♂ ♀								176 19				176 19	195
*Ph. mascittii*	♂ ♀		62 83										62 83	145
*Ph. papatasi*	♂ ♀				242 140	153 4	230 123	12386 1988	80 90	383 143			13474 2488	15962
*Ph. bergeroti*	♂ ♀							45 3					45 3	48
*Ph. sergenti*	♂ ♀		1277 293		173 99	210 4	361 112	2471 715	399	419 152			5310 1375	6685
*Ph. mongolensis*	♂ ♀									401 277			401 277	678
*Ph. alexandri*	♂ ♀				161 44	244 13	612 48	6339 2670	34	2			7392 2775	10167
*Ph. kazeruni*	♂ ♀							78 29					78 29	107
*Ph. chabaudi*	♂ ♀							187					187 0	187
*Ph. riouxi*	♂ ♀					10	83	5					98 0	98
*Sergentomyia minuta*	♂ ♀	1898 1374	243 693	1678 1329	1175 2198	324 367	4403 801	8268 10059					17989 16821	34810
*Se. antennata*	♂ ♀						735 462	669 1272					1404 1734	3138
*Se. fallax*	♂ ♀						1222 2058	7752 5588	54 280	16 29			9044 7955	16999
*Se. schwetzi*	♂ ♀												0 200	200
*Se. christophersi*	♂ ♀						332	81					413 0	413
*Se. clydei*	♂ ♀							94			88		182 0	182
*Se. tiberiadis*	♂ ♀								659 946				659 946	1605
*Se. africana*	♂ ♀							517 1857	1 1				518 1858	2376
*Se. taizi*	♂ ♀								3				0 3	3
*Se. silva*	♂ ♀											42 57	42 57	99
*Grassomyia dreyfussi*	♂ ♀							168 157	1				169 158	327
TOTAUX	♂	6190	8197	3143	11306	4817	8678	44767	1404	1221	88	42	89853	
	♀	2171	1815	1333	4668	388	3604	24810	1540	601	0	57	40987	
	♂+♀	8361	10012	4476	15974	5205	12282	69577	2944	1822	88	99	130840	

La ventilation des phlébotomes présents dans cette collection (Tableau I, Fig. 3 et 4) selon leurs 10 pays d'origine fait ressortir que plus de la moitié des échantillons prélevés proviennent du Maroc. Ceci n'est pas surprenant dans la mesure où J.-A. Rioux a travaillé durant 30 années dans ce pays qu'il affectionnait particulièrement [14, 19, 20, 21, 28, 40, 45, 46].

En Afrique du Nord, la collection est riche de nombreux spécimens provenant de deux pays qu'il a également beaucoup prospectés: la Tunisie [4, 24, 40] et l'Algérie [2, 11, 12, 23, 34, 47]. Dans ces derniers pays, les phlébotomes identifiés comme étant *Ph. chabaudi* ont été réexaminés et séparés en *Ph. chabaudi* et *Ph. riouxi* depuis la description de cette dernière espèce [8] et la validation de son statut taxinomique spécifique par des approches moléculaires classiques [3, 15] ou de dernière génération [5] malgré une publication erronée les considérant comme étant des espèces synonymes [48].

Les spécimens de France sont assez nombreux et furent pour certains parmi les premiers capturés et mis en collection [9, 29, 31, 32]. Le foyer cévenol de leishmaniose à *Leishmania infantum* constitue le foyer français historique qui fut le premier étudié par J.-A. Rioux [10, 13, 22, 38, 39, 41, 42, 43]. C'est à cette occasion qu'il comprit la notion de maladie à précellence vectorielle et l'importance de l'abondance maximale du vecteur selon l'altitude [30]. Plus de 10 000 phlébotomes de Corse sont également présents dans la collection [35, 36].

La collection des spécimens d'Espagne est abondante. Elle inclut des espèces peu abondantes telle *Ph. alexandri* [25], mais malheureusement pas de spécimen de *Ph. chabaudi* [26].

Dans le contexte géopolitique actuel complexe du Proche et du Moyen-Orient, les spécimens de la collection provenant de Syrie [44] et du Yémen [6] revêtent un intérêt tout particulier.

À l'exception de topotypes de *Ph. chabaudi* et de *Ph.* (*Lar.*) *chadlii* [37], il n'y a ni types ni topotypes de *Ph.* (*Lar.*) *mariae* [27] dans la collection.

Enfin, il est à noter que des phlébotomes capturés par J.-A. Rioux dans d'autres pays tels le Tchad [1] semblent perdus tandis que ceux capturés à Chypre [7] ou au Sultanat d'Oman [33] figurent dans d'autres collections.

## Conclusion

La collection de phlébotomes de J.-A. Rioux est actuellement en cours d'intégration à la Plateforme des centres de ressources biologiques de Reims (PF CRBs Reims), située au Pôle de biologie du CHU de Reims afin d’être stockée physiquement et informatiquement de manière sécurisée. À terme, toute la collection sera disponible pour la communauté scientifique auprès de la PF CRBs Reims. L'accès aux lames se fait par le site Internet de la PF CRBs Reims https://www.chu-reims.fr/offre-de-soins/centres-ressources/centre-ressources-biologiques-champagne-ardenne. Le formulaire de demande d’échantillon disponible sur le site internet doit être rempli et envoyé à l'adresse suivante: pfcrbs@chu-reims.fr. Chaque demande est soumise au Conseil scientifique de la PF CRBs Reims. Après examen, un devis et un contrat de mise à disposition sont proposés. À la signature des documents, les échantillons accompagnés de leurs données seront envoyés.

## Conflits D'intérêts

L'auteur déclare ne pas avoir de conflit d'intérêt.

## Remerciements

L'auteur remercie les enfants de Monsieur Rioux qui ont autorisé le transfert de la collection, conformément aux volontés de leur père. Il remercie également Mireille Cousinat qui a très activement participé à l'inventaire de cette collection ainsi que Camille Rocaboy et Isabelle Villena qui ont favorisé l'insertion de la collection dans la Plateforme des centres de ressources biologiques de Reims (PF CRBs Reims).
